# Evaluating enzyme replacement therapies for Anderson-Fabry disease: commentary on a recent report

**DOI:** 10.1590/1678-4685-GMB-2017-0345

**Published:** 2018-10-11

**Authors:** Roberto Giugliani, Stephanie Westwood, Hartmann Wellhoefer, Jörn Schenk, Andrey Gurevich, Christoph Kampmann

**Affiliations:** ^1^ Medical Genetics Service, HCPA, and Department of Genetics, UFRGS, Porto Alegre, Brazil; ^2^ Cognite, London, UK; ^3^ Shire, Zug, Switzerland; ^4^ Universitätsmedizin Mainz, Mainz, Germany

**Keywords:** Agalsidase alfa, agalsidase beta, Anderson-Fabry disease, enzyme replacement therapy

## Abstract

Anderson-Fabry disease (AFD) is a rare lysosomal storage disorder. Randomized controlled clinical trials (RCTs) are preferred as the highest category of evidence, but limited availability of robust evidence in rare diseases may necessitate the use of less rigorous evidence. An analysis of cohort studies of enzyme replacement therapies for AFD published in 2017 by El Dib and coworkers made treatment recommendations that contradict previously published findings from RCTs and a systematic Cochrane review. Our commentary outlines concerns regarding selection criteria and statistical methods with their analysis.

Anderson-Fabry disease (AFD) is a rare, inherited, lysosomal storage disorder with serious, progressive, systemic effects that frequently lead to premature mortality (MacDermot *et al.*, 2001a,b). Meta-analyses of high-quality randomized controlled clinical trials (RCTs) are generally considered the most rigorous category of evidence for the assessment of therapies (Barton, 2000; Day, 2010), but in rare diseases such as AFD, resource limitations and other practical considerations frequently limit their availability (Day, 2010). When evidence from high-quality RCTs is lacking, less rigorous evidence may be used, including results from observational studies and case reports, together with expert opinion (Day, 2010).

Comparative data for the two enzyme replacement therapies (ERTs) currently available for AFD, agalsidase alfa and agalsidase beta, are scarce and represented by the Canadian Fabry Disease Initiative, which is an ongoing, long-term, controlled, randomized, head-to-head clinical comparison of the two ERTs (Sirrs *et al.*, 2014), and a Cochrane systematic review of RCTs (El Dib *et al.*, 2016). These analyses found no notable differences in outcomes between the two ERTs (El Dib *et al.*, 2016; Sirrs *et al.*, 2014). In contrast to these reports, El Dib and coworkers subsequently published results of a pooled analysis of proportions from cohort studies and concluded that treatment with agalsidase beta should be recommended over agalsidase alfa (El Dib *et al.*, 2017). We are writing this commentary to raise our serious concerns about their methodological approach and validity of the conclusions. As these conclusions include overt recommendations about treatment management decisions, we feel that it is important to minimize potential misunderstandings among clinicians involved in the management of patients with AFD.

Although the stated objective of the El Dib *et al.* (2017) analysis was to evaluate the efficacy and safety of ERT for AFD, in our opinion, the authors used an inappropriate methodological approach that does not allow conclusions to be drawn about the relative efficacy of the two available ERTs for AFD. As noted above, the conclusions in the 2017 El Dib manuscript also contradict the findings published in 2016 by the same first author, using the Cochrane Institute’s systematic review process to analyze results from RCTs (El Dib *et al.*, 2016), as well as the results from the Canadian Fabry Disease Initiative. We believe it is important that the scientific community and practicing physicians should have a clear understanding that the 2017 El Dib publication does not apply the well-established Cochrane methodology (Higgins *et al.*, 2016), despite the mention of Cochrane in the article title. This inclusion of “Cochrane” in the article title may be misleading, as it suggests that the paper is a Cochrane review. A number of specific concerns relating to outcome measures in the 2017 El Dib analysis are detailed in Table 1.

**Table 1:** Specific comments relating to outcome measures described in the 2017 analysis by El Dib and coworkers.

Mortality analysis
Only one of the four agalsidase alfa studies included mortality as an endpoint (the others noted mortality in the context of safety). Although a value of n=309 is quoted by El Dib *et al.* (2017) for the total mortality analysis, there were 555 patients included in the safety population alone of the Mehta *et al.* (2009) paper, which included mortality assessments. There is a considerable difference in the duration of the selected studies; because the agalsidase alfa studies were generally of longer duration, more mortality events would be anticipated in this group. One of the selected agalsidase alfa studies evaluated patients with end-stage renal disease (Pastores *et al.*, 2007), who might be expected to have more mortality events. The analysis included neither the largest (n=677) study of agalsidase alfa that specifically looked at mortality (Beck *et al.*, 2015), nor the mortality data from the Canadian Fabry Disease Initiative (Sirrs *et al.*, 2014).
Renal outcomes analysis
Only one agalsidase beta study was included in the renal outcomes analysis. Only one of the five agalsidase alfa studies included renal events as an endpoint, and two of the studies did not include information on dialysis or transplantation. The Fabry Outcome Survey study/cohort included in the El Dib analysis provided a descriptive analysis of renal events in only 78 adult patients receiving agalsidase alfa (Barba-Romero *et al.*, 2011), whereas more detailed renal endpoint data (including initiation of dialysis and renal transplantation) are available in a separate analysis of a larger population of patients from the same data source (Mehta *et al.*, 2009).
Cardiac outcomes analysis
The comparability of endpoints was not always clear (e.g., three of the cardiac “events” reported by McKechnie *et al.* (2015) were not specified). The analysis did not take into account the different proportions of patients with left ventricular hypertrophy (LVH) at baseline, which can increase the probability of a cardiac event.
Cerebrovascular outcomes analysis
The analysis included a study in which three of the five women who experienced a stroke had a history of stroke before initiating agalsidase alfa (Whybra *et al.*, 2009). The analysis did not take into account the different proportions of patients with LVH at baseline, which can increase the probability of a cerebrovascular event.
Composite outcomes analysis
The analysis included mixed patient populations, including patients with end-stage renal disease, on dialysis, with LVH etc., who might be more likely to have an event. The analysis did not include the largest (n=677) study of agalsidase alfa that specifically looked at mortality (Beck *et al.*, 2015). The longer duration of the agalsidase alfa studies (up to 15 years) means that more events might be anticipated in this group. Although it was reported that the Germain *et al.* (2013) study had a follow-up of 9.5 years, the mean follow-up in this study was 4.8 years. The graph shows that two of the agalsidase alfa studies may have disproportionately affected the composite endpoint rates. One of the cited agalsidase beta studies does not appear to be included in the composite event rate graph (El Dib *et al.*, 2017).

The authors included 77 cohort studies (represented by 135 individual references) involving 15,305 participants in their “qualitative synthesis” (El Dib *et al.*, 2017). The majority of the included studies (79.2%; n=61) were available as full-text articles and 20.8% (n=16) were reported only in abstract form. Although the original authors, including Christoph Kampmann (one of the authors of this current commentary), were contacted in cases of multiple publications from the same study, only nine responses are included in the supporting information provided with the El Dib *et al.* (2017) publication. Of these nine responses, potential overlap in patient populations was identified by six of the original authors (covering a total of 23 publications). Although some information is provided about how such potential duplicate reporting of patient data was addressed, the selection of studies to be included appears to have been driven by practical rather than scientific or statistical considerations (e.g., numbers of patients reported, availability of full-text reports).

In the Results section of the abstract (El Dib *et al.*, 2017), it is stated that “77 cohort studies involving 15,305 participants proved eligible”. However, in the “Strengths and Limitations” section of the Discussion section, it is clarified that “out of the 77 cohort studies we were only able to include data in the meta-analysis from 39 (50.6%).” The proportion quoted (50.6%) relates to the number of studies/cohorts (i.e., 39 out of 77), whereas it would have been more informative to state the proportion of patients (rather than studies/cohorts) included in the quantitative analysis. The number of patients (and their baseline characteristics) in the 39 included studies is unknown.

In contrast to their initial Cochrane review (El Dib *et al.*, 2016), which focused on data from RCTs, the authors specifically excluded these studies from the 2017 analysis (El Dib *et al.*, 2017). Data from RCTs are more likely to be clinically robust than those from nonrandomized studies/cohorts, which are the focus of the 2017 El Dib analysis. Important and relevant safety parameters (such as infusion-related reactions and the development of antidrug antibodies) were also excluded from the analysis.

The supporting information provided with the El Dib *et al.* (2017) paper illustrates the considerable variation in inclusion and exclusion criteria between the selected cohort studies. The pooling of results from this highly heterogeneous population is of questionable value and makes it extremely difficult to apply the overall conclusions from the analysis to specific patient groups. For example, the different studies/cohorts included variable proportions of patients who might be expected to be at increased risk for AFD-related events, such as patients with end-stage renal disease or with a history of stroke prior to treatment. It would have been useful if the authors had provided more detailed information on each study/cohort selected, in particular the precise number of patients concerned plus a brief description of the study design. Another variable that is not included in the analysis is the date when the individual studies/cohorts started – this is also likely to have an impact on patient outcomes, as standards of care continue to evolve.

Although the authors state that 39 studies/cohorts were included in their quantitative synthesis (El Dib *et al.*, 2017), data from only 16 were used in the comparisons of agalsidase beta (maximum of four studies/cohorts) and agalsidase alfa (12 studies/cohorts). Given the number of studies included, there is clearly the potential for selection bias in the comparisons of efficacy based on the four outcomes considered (all-cause mortality, renal complications, cardiovascular complications, and cerebrovascular complications), with only four studies/cohorts used to demonstrate the efficacy of agalsidase beta versus 12 studies/cohorts for agalsidase alfa (three versus nine, if pediatric studies are excluded). In addition, the largest real-world analysis to date examining renal, cardiac, morbidity, and mortality outcomes from over 600 patients (Beck *et al.*, 2015) was not included in the 2017 El Dib analysis, raising the question of selection bias.

Furthermore, the studies/cohorts used to demonstrate the efficacy of agalsidase beta (El Dib *et al.*, 2017) included one large study (n=1044 patients) and two very small studies (n=9), plus a pediatric study of 15 patients, which does not allow a relevant analysis of heterogeneity. For example, for the renal complications outcome measure only the large study is used, which automatically prevents the calculation of the I^2^ measure of heterogeneity. By contrast, the size of the studies/cohorts used to demonstrate the efficacy of agalsidase alfa ranged from seven to 336 patients (average of 79 patients, excluding the three pediatric studies), which allowed the heterogeneity of the data involved in this pooled analysis of proportions to be estimated.

The authors describe their analysis as a “proportional meta-analysis” in the Methods section (El Dib *et al.*, 2017). The correct terminology for this type of analysis (as used in the title and abstract) is “pooled analysis of proportions.” The results obtained from this pooled analysis of proportions are based on aggregate data, which are less desirable than patient-level data. The use of aggregate data in this instance limits the analysis performed for the “simple linear regressions and analysis of variance”, as this type of regression analysis is only informative when the effect of potential confounding factors can be assessed, which was not the case here.

Asymmetry or gaps in the mortality plots included in the publication El Dib *et al.* (2017) are suggestive of bias, most often due to studies that are smaller, have nonsignificant results, or have an effect in the opposite direction from the others or from that expected. The small-study effect can occur when small studies have systematically different effects from the large ones. The influence of the small-study effect on the results of the pooled analysis appears in mortality plots for agalsidase alfa and untreated patients, showing clear between-study heterogeneity (I^2^ > 0), which is not apparent in the plot for agalsidase beta owing to: (i) the greater weight of at least one study and (ii) the comparatively small number of studies. The impact of the follow-up period in the analysis was not described (nondifferential).

It should also be noted that although the results in the El Dib *et al.* (2017) publication show a discordant effect for groups of studies in three mortality plots, no explanation of variance is included. Conventionally, sources of variation should be identified and their impact on effect size should be quantified using statistical tests and methods, such as analysis of variance or weighted meta-regression. Furthermore, when high heterogeneity is evident, individual data should be not pooled and definitive conclusions should only be drawn once more studies become available.

Although the authors planned to perform sensitivity analysis by sex, age (adults versus children), follow-up period (< 5 years versus ≥ 5 years), and AFD phenotype (classical versus nonclassical), only the analyses by age and follow-up period were included, as there were insufficient studies to allow analysis of the other two variables (El Dib *et al.*, 2017). However, among the 77 selected studies/cohorts, 62 (> 80%) included both male and female patients, so it is surprising that the analysis by sex was not included.

In conclusion, we consider the methodology applied in the El Dib *et al.* (2017) to be inadequate. The numerous limitations of this analysis undermine the conclusions drawn by the authors. As these conclusions extend to making recommendations for management strategies for patients with AFD, the methodological shortcomings of the analysis underpinning these recommendations need to be highlighted, so that clinicians have a clear understanding of the data relating to the relative efficacy of available ERTs for AFD.
